# Real-time tumor tracking for magnetic resonance-guided radiotherapy using label-efficient foundation model adaptation^☆^

**DOI:** 10.1016/j.phro.2026.101078

**Published:** 2026-09-10

**Authors:** Amparo S. Betancourt Tarifa, Marcel Verheij, René Monshouwer, John J. Hermans, Peter J. Koopmans, Erik van der Bijl

**Affiliations:** a Department of Radiation Oncology, Radboud University Medical Center, Nijmegen, The Netherlands; b Diagnostic Image Analysis Group, Department of Medical Imaging, Radboud University Medical Center, Nijmegen, The Netherlands; c Department of Medical Imaging, Radboud University Medical Center, Nijmegen, The Netherlands

**Keywords:** Deep learning, Foundation models, MRI-linac, MR-guidance, Tumor tracking

## Abstract

**Background and Purpose:**

Real-time tumor tracking on cine magnetic resonance imaging (cine-MRI) enables intra-fraction motion management in magnetic resonance-guided radiotherapy. Pipelines often rely on deformable registration or template matching to propagate contours, which can degrade under non-rigid motion and cine-MRI artifacts. This study evaluated two MedSAM2-based adaptations for real-time tracking with limited labels.

**Materials and Methods:**

The TrackRAD2025 dataset includes sagittal cine-MRI sequences from six institutions acquired on 0.35 T and 1.5 T MRI-linear accelerators, with 50 labeled and 477 unlabeled training cases and 50 test cases. Tracking was formulated as frame-wise target segmentation from the first-frame ground-truth mask. Method A fine-tuned MedSAM2 on 40 labeled cases and used rank-weighted checkpoint averaging. Method B combined two MedSAM2 models trained on different labeled splits augmented with pseudo-labels, and merged their predictions during inference.

**Results:**

On the hidden test set, Method A ranked highest on Dice similarity coefficient (DSC), center distance (CD), 95th-percentile Hausdorff distance (HD95), and mean average surface distance (MASD), with DSC = 0.891, CD = 1.47 mm, HD95 = 4.22 mm, MASD = 1.66 mm, relative dose to 98% of the target volume ($D_{98\text{\%}}$) = 0.936, and runtime of 0.04 s per frame. Method B achieved similar geometric performance and numerically higher relative $D_{98\text{\%}}$ (0.953), at 0.10 s per frame. Paired Wilcoxon signed-rank testing showed no significant differences after correction for multiple testing.

**Conclusions:**

MedSAM2 adaptation enabled real-time cine-MRI tumor tracking with limited labels. The single-model approach provided faster inference and simpler deployment, while semi-supervised ensembling showed no significant improvement at higher computational cost.

## Highlights

• Real-time tumor tracking was feasible with limited labeled cine imaging data.
• The fine-tuned foundation model achieved a Dice score of 0.891 on hidden data.
• Checkpoint averaging gave a single-model solution running at 0.04 s per frame.
• Most residual errors were boundary-related rather than complete tracking loss.

## Graphical abstract

**Figure:**
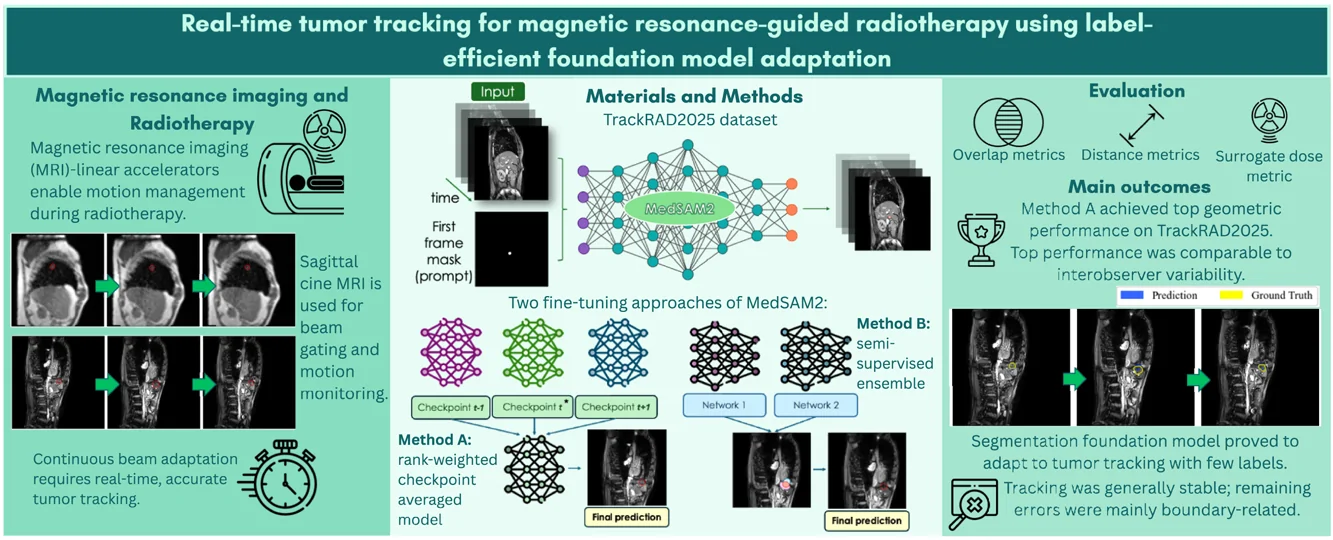

## 1 Introduction

Magnetic resonance-guided radiotherapy (MRgRT) provides real-time soft-tissue visualization during irradiation, enabling intra-fraction motion management and potentially tighter treatment margins [1]. Motion management is especially relevant for abdominal and thoracic targets affected by respiration, but also for detecting intra-fraction target drift. Accurate tracking and gating can support margin reduction and thereby decrease exposure of nearby organs at risk while maintaining target coverage.

A pragmatic clinical workflow uses 2D+t cine-MRI for beam gating, where irradiation is paused when the target moves outside a predefined boundary or tracking confidence drops [2], [3]. While gating can improve geometric precision, it reduces beam-on efficiency and prolongs treatment time. This motivates interest in continuous contour adaptation approaches such as multi-leaf collimator (MLC) tracking, which require accurate low-latency tracking [4], [5]. Current solutions often rely on template matching or deformable image registration (DIR) to propagate contours from an annotated frame, but they can degrade under large non-rigid motion, appearance changes, and cine-MRI artifacts [6].

Deep learning methods shift most of the computation time to the training phase and provide fast inference, making them attractive for real-time tracking [7]. However, until recently, the field lacked a large public multi-institutional dataset and standardized evaluation to compare methods fairly. The TrackRAD2025 challenge [8] addresses this gap by providing a public benchmark for real-time tumor tracking on time-resolved sagittal 2D cine-MRI across centers, anatomies, and acquisition settings.

Promptable segmentation foundation models have emerged as an alternative to task-specific pipelines. The Segment Anything Model 2 (SAM2) introduced a streaming-memory video segmenter for prompt-conditioned mask propagation [9], and MedSAM2 adapted this paradigm to medical imaging and video, demonstrating strong cross-domain generalization [10].

This study evaluated two MedSAM2-based strategies for the TrackRAD2025 first-frame-prompted cine-MRI tracking task. Method A used labeled-data fine-tuning with checkpoint averaging, whereas Method B added pseudo-labeled supervision and ensembling. The aim was to compare accuracy, runtime, scanner- and anatomy-wise performance, temporal failure modes, and whether the pseudo-label-augmented ensemble improved over the labeled-only approach.

## 2 Materials and methods

### 2.1 Dataset

This study used the multi-institutional TrackRAD2025 cine-MRI dataset [11], developed for benchmarking real-time target tracking in MRgRT. It consists of sagittal 2D cine-MRI sequences acquired during treatment on 0.35 T MRIdian systems (ViewRay, USA) and 1.5 T Unity systems (Elekta, Sweden), and includes thoracic, abdominal, and pelvic targets from six international centers.

The dataset comprised 585 cases, 50 labeled and 477 unlabeled training cases, an 8-case preliminary hidden test set, and a 50-case final hidden test set. The final hidden test set included cases from centers A, B, C, D, and X. Labeled training cases came from a subset of institutions, whereas the hidden test set included additional institutions, introducing an institutional distribution shift. Targets were annotated per frame and combined using STAPLE [12] when multiple observers were available.

Image intensities were clipped to the 0.5th–99.5th foreground percentiles, min–max normalized, and upsampled from 270 × 270–450 × 450 pixels to 512 × 512 pixels. Images were resampled with bilinear interpolation and masks with nearest-neighbor interpolation.

All models were developed during the TrackRAD2025 challenge using only the public training set, while performance was assessed exclusively through the hidden-test challenge evaluation system.

For model selection and ablations, the public labeled set was divided into patient-wise internal training/validation splits stratified by anatomical region and imaging center. These included a standard 80/20 split and a center-held-out split to reflect the expected institutional distribution shift; both were used only during development.

**Fig. 1:**
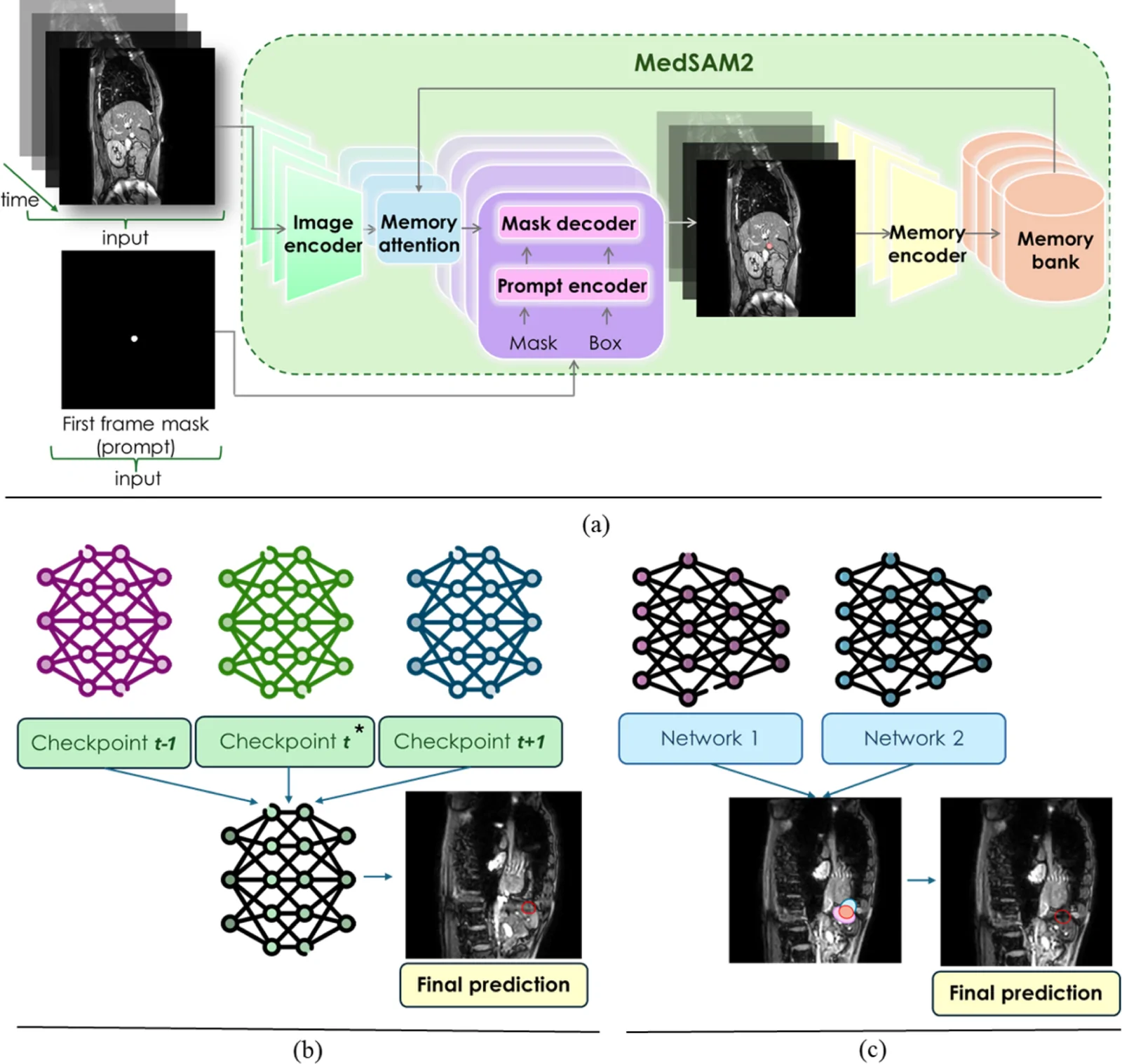
Overview of the MedSAM2-based cine-MRI tumor tracking framework and the two model consolidation strategies. (a) MedSAM2-based cine-MRI tracking pipeline. The manually annotated mask from the first frame is used as a prompt to guide segmentation in subsequent frames. Current-frame image features are combined with temporal information through memory attention, while prior predictions are encoded into a persistent memory bank to support tracking over time. (b) Schematic of Method A (model soup). A single training trajectory is used, and the best-performing checkpoint is averaged with its neighboring checkpoints to obtain one consolidated model, which is then used for final prediction. (c) Schematic of Method B (ensemble). Multiple models are trained independently and their predictions are combined at inference time to produce the final ensemble output.

### 2.2 Model architecture

Both proposed tracking solutions build on MedSAM2, a promptable segmentation foundation model derived from SAM2 and adapted to medical imaging. MedSAM2 consists of an image encoder, a prompt encoder, a mask decoder, and a memory mechanism that allows information from previously processed frames to be reused when segmenting the current frame (Fig. 1a).

Each cine-MRI sequence was processed sequentially in time. The first frame and its corresponding tumor mask were used for initialization. Subsequent frames were processed one at a time. For each frame, the predicted mask and image features were encoded into a memory representation and added to a fixed-capacity first-in-first-out (FIFO) memory bank. This bank retained the prompted first frame together with fused image–mask embeddings from six recent frames, following the default MedSAM2 configuration. Together with the current query frame, this provided temporal context from up to eight frames.

### 2.3 Training setup

This study compared two approaches to combine multiple trained model states. In Method A, high-performing checkpoints from a single training run (i.e. saved model states from different training epochs) were combined through rank-weighted averaging, or *model soup* [13], yielding a single final network without inference-time overhead (Fig. 1b). Method B combined pseudo-labeled supervision with model diversity through two independently trained models, B1 and B2, using different labeled-data splits augmented with curated pseudo-labels. Their predictions were combined during inference (Fig. 1c).

Both methods used the MedSAM2 reference setup with the SAM2.1 Hiera-tiny backbone. Training used batch size of 1 and AdamW optimization [14], with 8-frame clips for Method A and 10-frame clips for Method B. For Method A and Model B1, the base and image-encoder learning rates were $2\times 10^{-5}$ and $1\times 10^{-5}$, respectively; Model B2 used $2.5\times 10^{-5}$ and $1.5\times 10^{-5}$. Learning rates followed cosine decay. The loss combined Focal, Dice, intersection over the union (IoU), and object-score terms with weights of 20, 1, 1, and 1. Augmentation comprised random horizontal flipping, random affine transformations, color jitter, and random grayscale conversion. The remaining MedSAM2 defaults were retained. During development, configuration choices not central to the definitions of Method A and Method B, including ensemble composition, were selected on internal validation before hidden-test evaluation. All training was initialized from the MedSAM2 authors’ latest checkpoint (MedSAM2_latest.pt). Code and model weights are publicly available on Zenodo [15].

**Method A (model soup)**

Method A was fine-tuned for 15 epochs on an internal split of 40 training and 10 validation cases, with the validation set used for checkpoint selection. The training duration was empirically selected based on internal validation performance.

The rank-weighted model soup was constructed by averaging the model parameters of the best validation checkpoint and its two nearest temporal neighbors. Let $C=\left\{c_{t}^{*},c_{t-1},c_{t+1}\right\}$ denote the selected checkpoint set, where $c_{t}^{*}$ is the best validation checkpoint and $c_{t-1}$ and $c_{t+1}$ are the previous and next checkpoints in training time. Each checkpoint c∈C was assigned a validation rank r(c)∈{1,2,3}, with lower ranks indicating better validation performance. The normalized weight for checkpoint c was defined as $w\left(c\right)=\left(1/r\left(c\right)\right)/\sum_{c^{\prime }\in C}\left(1/r\left(c^{\prime }\right)\right)$. The final checkpoint-averaged parameters $\overset{¯}{\theta }$ were then obtained as the weighted average of the checkpoint parameters $\theta^{\left(c\right)}$, as shown in Eq. (1).

(1) $$\overset{¯}{\theta }=\underset{c\in C}{\sum }w\left(c\right)\theta^{\left(c\right)}.$$

The checkpoint-averaged model was selected prospectively during the challenge, before access to hidden-test labels, based on validation ranking and the expectation that averaging neighboring checkpoints could reduce checkpoint-specific variability [13], [16].

**Method B (ensemble)**

Method B combined two MedSAM2 models trained on different internal splits of the labeled data and augmented with curated pseudo-labeled sequences. Model B1 used the standard 80/20 split, whereas Model B2 used the center-held-out split. To increase temporal diversity, additional training samples were generated by temporally subsampling the labeled sequences (every 2nd and 4th frame).

Model B1 was trained for 30 epochs and the best validation checkpoint was retained. Model B2 was trained for 45 epochs and consolidated via a rank-weighted model soup (as in Method A). This asymmetric choice was intended to increase model diversity.

At inference, Models B1 and B2 were run separately and their outputs were combined before generating the final mask. Specifically, the raw prediction scores from both models were averaged, converted into a probability map, and thresholded to produce the final segmentation.

**Unlabeled data curation and pseudo-label generation**

**Fig. 2:**
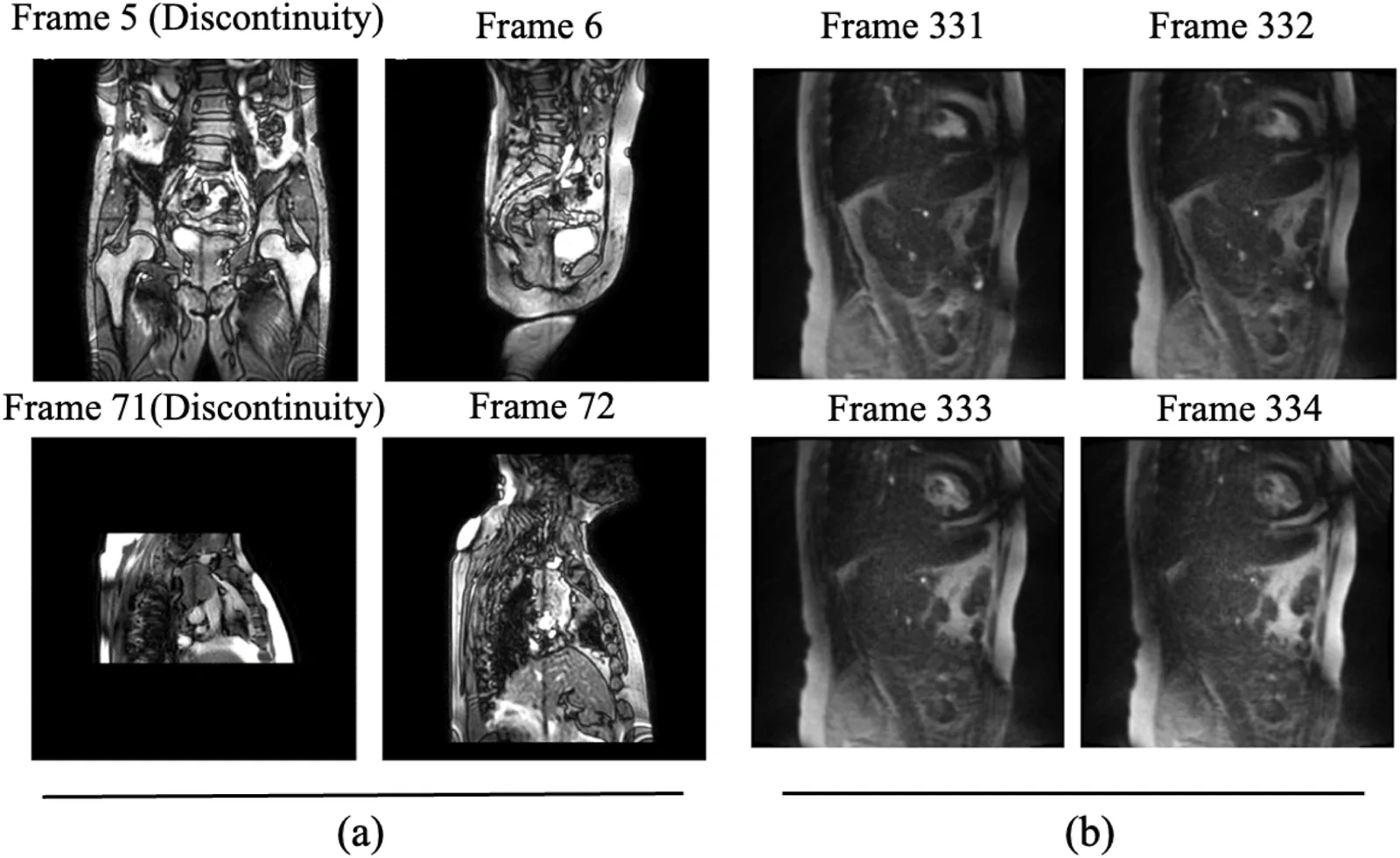
Examples of frames excluded during unlabeled-data curation. (a) Elekta Unity (1.5 T): detected temporal discontinuities; each row corresponds to a different sequence and shows the flagged frame with its neighboring frame. (b) ViewRay MRIdian (0.35 T): frames with degraded image quality during gantry motion, resulting in reduced visibility of anatomy.

To increase training data for Method B, unlabeled sequences were pseudo-labeled through a scanner-specific pipeline. Quality control first removed temporal discontinuities in Elekta Unity sequences and degraded frames in ViewRay MRIdian sequences (Fig. 2), excluding sequences with excessive frame removal. Scanner-specific 2D nnU-Net models [17], trained on the labeled TrackRAD2025 cases to segment the challenge target structure, then generated frame-wise initial pseudo-labels, which were filtered using fold-consensus uncertainty and iterative retraining [18]. For retained sequences, the accepted first-frame nnU-Net pseudo-label initialized MedSAM2, and an ensemble of the three best Method A checkpoints propagated masks across the sequence. After visual review, 296/477 unlabeled sequences were used as additional supervision for Method B.

**Fig. 3:**
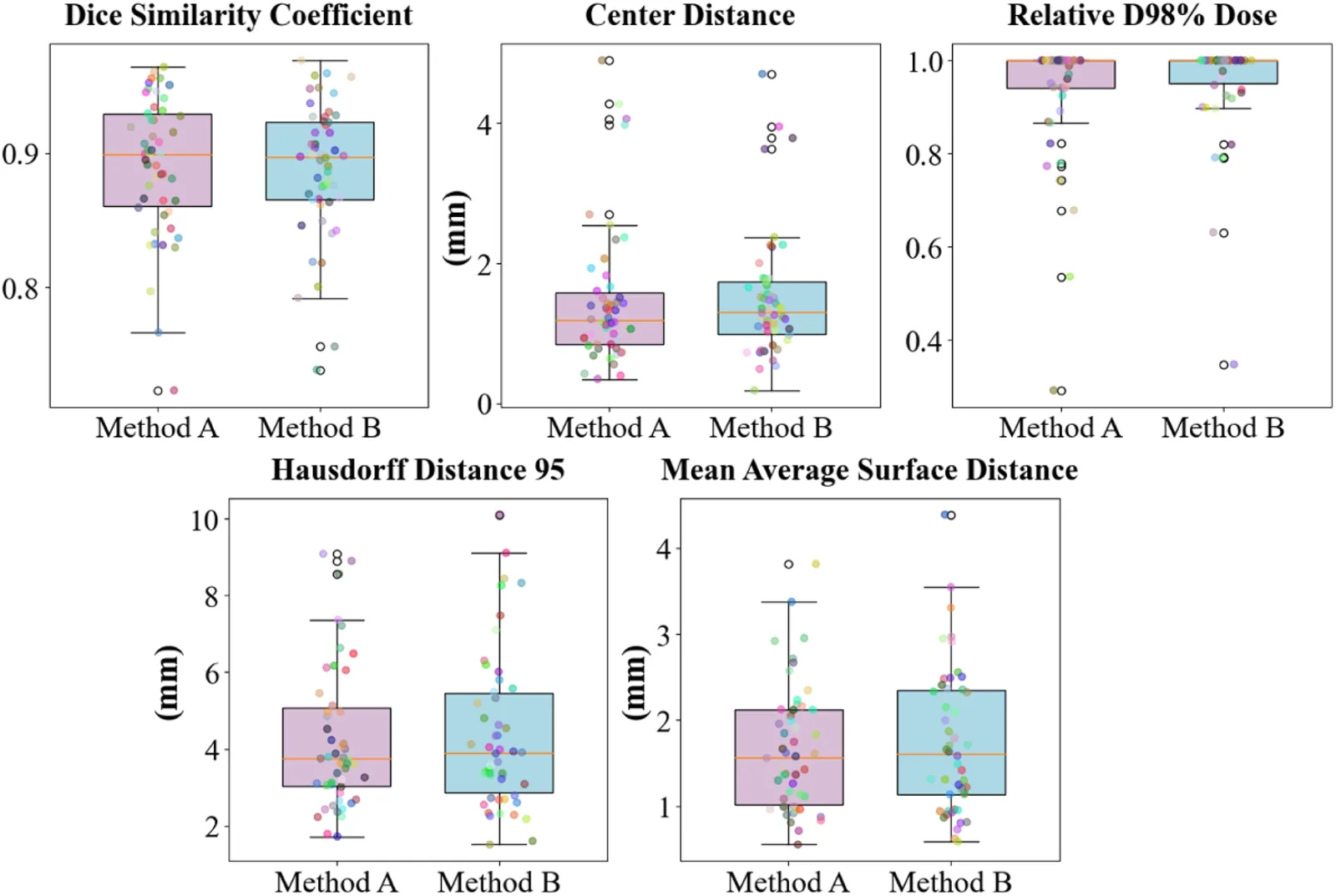
Distribution of per-case performance on the hidden final test set (50 cases) for Method A (model soup) and Method B (ensemble). Boxplots show median and interquartile range (IQR) with whiskers extending to 1.5×IQR; points indicate individual cases. Metrics include DSC, CD, $D_{98\text{\%}}$, HD95, and MASD.

### 2.4 Evaluation

The TrackRAD2025 challenge platform was used to evaluate the hidden final test set using DSC, HD95, MASD, CD, relative $D_{98\text{\%}}$, and runtime [8]. Relative $D_{98\text{\%}}$ was defined as the ratio between gross tumor volume (GTV) $D_{98\text{\%}}$ under a centroid-error-shifted surrogate dose distribution, simulating multi-leaf collimator tracking, and the corresponding ground-truth dose distribution [19]. Submissions were executed on a single NVIDIA A10G GPU (24 GB); runtime per frame excluded initialization overhead, and methods exceeding 1 s per frame were ineligible for ranking. Although the leaderboard reported only the highest-ranked submission per team, results for both proposed methods are presented in this study, together with the next two highest-ranked official submissions for comparison.

### 2.5 Statistical analysis

Statistical analyses were performed at the case level using paired per-case metrics. Paired comparisons used two-sided Wilcoxon signed-rank tests. Mean paired differences were reported with unadjusted 95% percentile bootstrap confidence intervals based on 10,000 paired case-level resamples. Holm correction was applied to the Wilcoxon p-values across the five metrics for Method A versus Method B and model soup versus single checkpoint, and across the ten comparisons of Methods A and B with zero-shot MedSAM2.

### 2.6 Temporal failure analysis

To better characterize temporal failure modes, an exploratory frame-wise analysis was performed after the challenge using Method A predictions from the 30 publicly released test sequences. The remaining 20 cases, including center D, were unavailable; therefore, this subset may not fully represent the complete test set.

Moderate and severe adverse frames were defined as DSC <0.80 and <0.70, HD95 >5 and >10 mm, MASD >2 and >4 mm, and CD >3 and >5 mm. Adverse-frame burden and consecutive runs were quantified, with run durations converted to seconds using sequence-specific frame rates.

Sensitivity analyses used adverse-direction sequence-specific bounds (mean ±kSD; k=1,2), restricted to SD ≥0.05 for DSC or SD ≥1 mm for distance metrics.

Substantial centroid error was defined as 95th-percentile CD >5 mm. Relative $D_{98\text{\%}}$ was compared between sequences with and without substantial centroid error using a two-sided Mann–Whitney U test, and its association with 95th-percentile CD was assessed using Spearman correlation.

## 3 Results

On the hidden final test set, Method A achieved the highest geometric performance among the compared methods, with DSC of 0.891, CD of 1.47 mm, HD95 of 4.22 mm, and MASD of 1.66 mm, while retaining real-time inference at 0.04 s/frame (Table 1). Method B achieved similar geometric performance and a numerically higher relative $D_{98\text{\%}}$ score on the centroid-shift-based surrogate dose metric (0.953 vs. 0.936), at the cost of higher runtime (0.10 s/frame). On the preliminary hidden test set, Method A showed a similar advantage over Method B (DSC, 0.917 vs. 0.913; CD, 1.60 vs. 1.66 mm; HD95, 3.97 vs. 4.20 mm; MASD, 1.41 vs. 1.44 mm; relative $D_{98\text{\%}}$, 0.96 vs. 0.95), although its small size limited interpretation.

Per-case performance distributions on the hidden final test set are shown in Fig. 3. Median DSC was 0.900 (IQR, 0.859–0.930) for Method A and 0.897 (IQR, 0.865–0.925) for Method B, while relative $D_{98\text{\%}}$ was below 0.90 in 10/50 and 8/50 cases, respectively.

**Table 1:** Performance on the hidden final TrackRAD2025 test set (50 cases). For Methods A and B and the post-hoc ablations, values are reported as mean [IQR]; for the other official submissions, only challenge-reported aggregate values were available. Ranks in parentheses are metric-specific ranks among the four listed official submissions, not the full leaderboard. Best values are shown in bold. Arrows indicate metric direction: ↑ higher is better and ↓ lower is better.

**Method**	**DSC**↑	**CD [mm]**↓	$D_{98\text{\%}}$↑	**HD95 [mm]**↓	**MASD [mm]**↓	**Runtime [s]**↓

Method A (model soup)	**0.891** (1) **[0.859–0.930]**	**1.47** (1) **[0.83–1.62]**	0.936 (3) [0.941–1.000]	**4.22** (1) **[2.97–5.12]**	**1.66** (1) **[1.00–2.12]**	0.04 (2)
Method B (ensemble)	0.888 (2) [0.865–0.925]	1.50 (3) [0.96–1.75]	0.953 (2) [0.945–1.000]	4.38 (2) [2.77–5.51]	1.74 (2) [1.09–2.36]	0.10 (4)
Official #2 (SAM2)	0.886 (3)	1.49 (2)	**0.963** (1)	4.50 (3)	1.86 (3)	0.06 (3)
Official #3 (CoTracker)	0.876 (4)	1.66 (4)	0.925 (4)	4.54 (4)	1.92 (4)	**0.02** (1)

*Post-hoc ablations*						
Method A (single checkpoint)	0.891 [0.859–0.930]	1.48 [0.83–1.64]	0.937 [0.942–1.000]	4.22 [2.97–5.15]	1.66 [1.00–2.12]	–
MedSAM2, no fine-tuning	0.865 [0.799–0.925]	2.06 [0.97–2.29]	0.870 [0.837–1.000]	5.01 [3.30–6.24]	1.95 [1.14–2.59]	–

Mean paired differences, defined as Method A minus Method B, were ＋0.0030 for DSC (95% CI, −0.0018 to 0.0074), −0.16 mm for HD95 (95% CI, −0.34 to 0.01 mm), −0.08 mm for MASD (95% CI, −0.14 to −0.01 mm), −0.03 mm for CD (95% CI, −0.11 to 0.05 mm), and −0.017 for relative $D_{98\text{\%}}$ (95% CI, −0.033 to −0.004), with the latter indicating numerically higher values for Method B. None of the differences remained statistically significant after Holm correction (adjusted p=0.20–0.45).

**Fig. 4:**
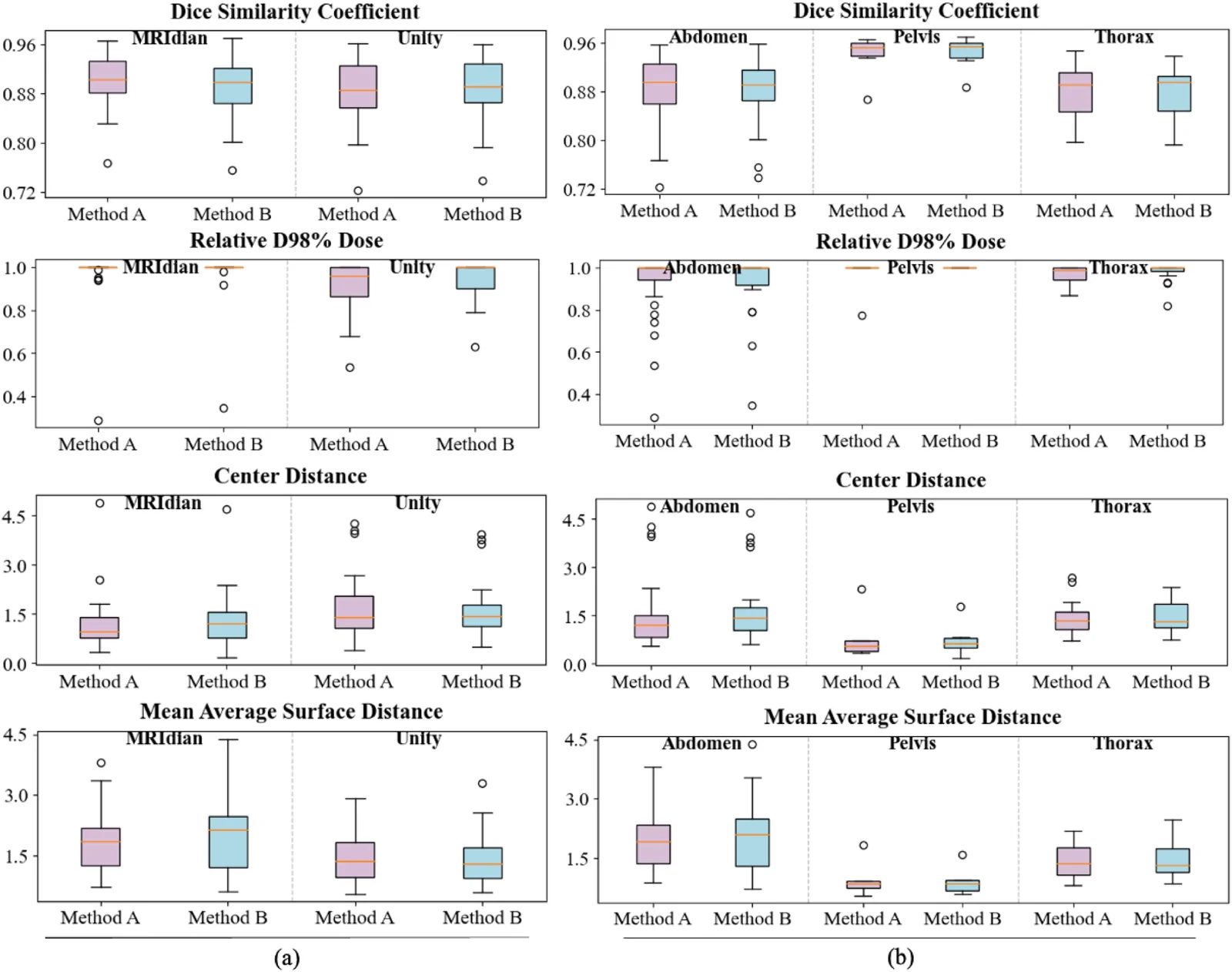
Per-case performance on the hidden final test set stratified by (a) scanner type and (b) anatomical region. (a) 0.35 T MRIdian systems (ViewRay, USA; centers A and D; n = 25) versus 1.5 T Unity systems (Elekta, Sweden; centers B, C and X; n = 25). (b) Abdomen (n = 30), pelvis (n = 5), and thorax (n = 15) subsets. Boxplots show median and IQR, with whiskers extending to 1.5×IQR.

Post-hoc ablation results are reported in Table 1. Relative to the zero-shot MedSAM2_latest.pt baseline, Method A significantly improved all five metrics after correction, including DSC (＋0.026) and HD95 (−0.79 mm; all adjusted p≤0.026). For Method B, only the improvement in relative $D_{98\text{\%}}$ remained significant (＋0.084; adjusted p=0.017). In contrast, the rank-weighted model soup did not significantly outperform the single best Method A checkpoint (adjusted p=0.29–1.00), indicating that task-specific fine-tuning, rather than checkpoint averaging, was the main source of improvement.

**Fig. 5:**
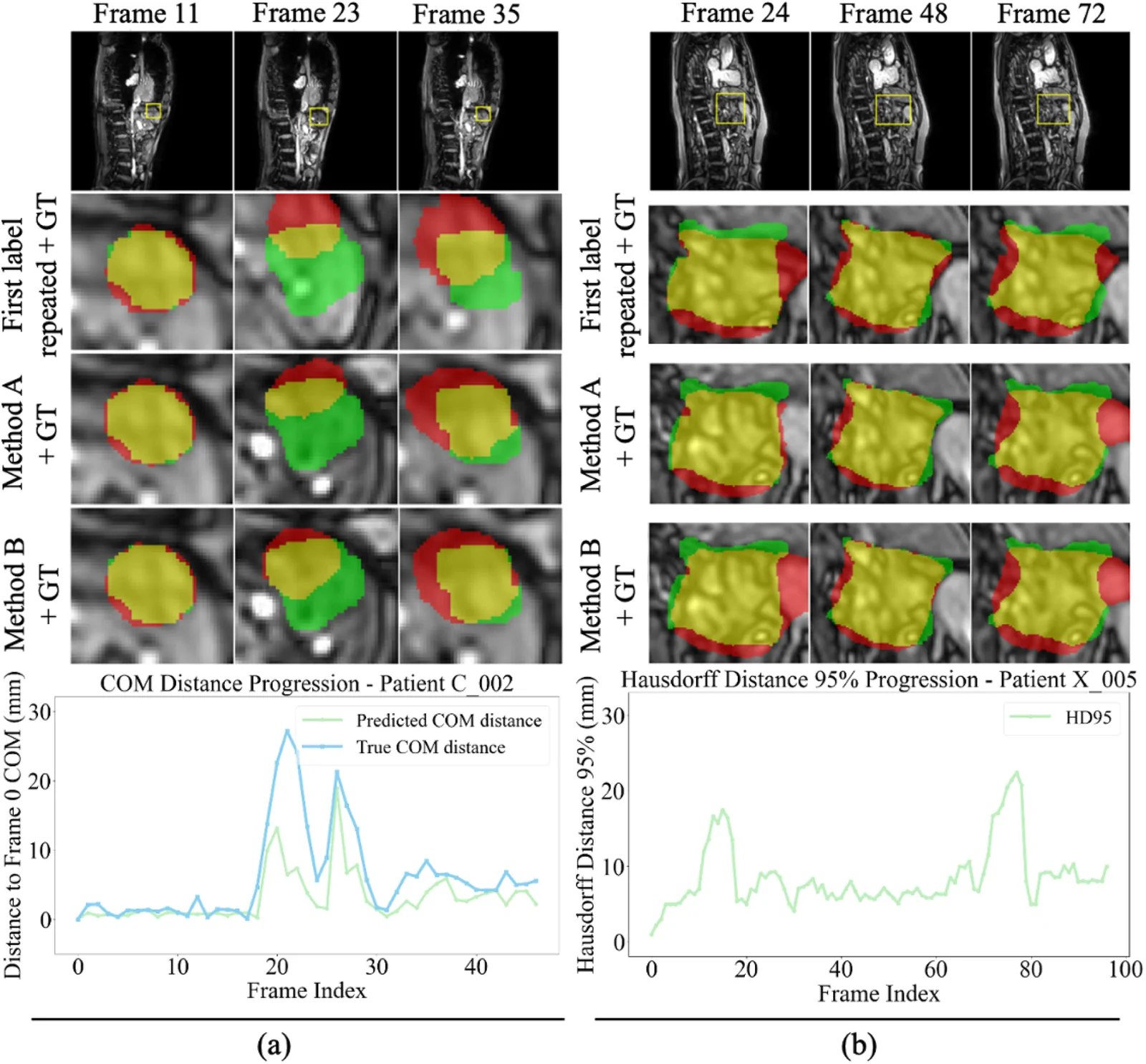
Representative temporal failure patterns in low-performing sequences. Columns show different time points of the same cine-MRI sequence. Top row: full field-of-view with the target indicated by a bounding box. Rows two to four: zoomed crops showing agreement between the ground truth (GT) and the repeated first-frame label, Method A (model soup), and Method B (ensemble), respectively, with yellow denoting overlap, green GT-only pixels (false negatives), and red prediction-only pixels (false positives). Bottom row: frame-wise error summary. (a) Transient degradation, where the segmentation drifts from the tumor over a few frames and then recovers; the plot shows the center-of-mass (COM) distance to the first-frame COM for prediction and GT. (b) Persistent boundary inaccuracy, where the tumor remains localized but contour errors persist along the borders; the plot shows frame-wise HD95.

Fig. 4 shows performance stratified by scanner type and anatomical region. Performance was broadly similar on cases acquired with 0.35 T MRIdian and 1.5 T Unity systems, with comparable overlap and center localization, slightly more favorable surface-based accuracy for 1.5 T cases, and no strong method-by-domain interaction. For relative $D_{98\text{\%}}$, most cases from both scanner groups were close to 1.0, although 1.5 T cases showed a broader lower tail and 0.35 T cases included a few more extreme outliers. Region-wise, pelvis cases were the easiest subset, abdomen cases the most challenging, and thorax cases showed intermediate behavior.

The temporal failure analysis showed that adverse frames were relatively common across the 30 released sequences but were generally brief. HD95 >5 mm occurred in 20/30 sequences and DSC <0.80 in 14/30. Median HD95 adverse-frame burden was 3.0% (IQR, 0%–25%) and exceeded 10% in 13/30 sequences versus 9/30 for DSC. Severe deviations were less frequent (HD95 >10 mm in 7/30; DSC <0.70 in 4/30). Adverse HD95 runs had a median duration of 0.81 s across the per-sequence mean run durations, although a few sequences showed prolonged errors. Relative thresholds identified adverse frames in nearly all sequences passing the variability filter and were therefore used only in sensitivity analyses.

Two main failure patterns were observed. The first was a transient localization error, where the segmentation moved away from the target and recovered (Fig. 5a). The second was recurrent boundary inaccuracy, where the target remained broadly localized but contour errors persisted along parts of the boundary (Fig. 5b). Substantial centroid error occurred in 6/30 sequences and was associated with lower relative $D_{98\text{\%}}$ (median, 0.761 vs. 1.000; U=4, p<0.001). Across all sequences, 95th-percentile CD was strongly negatively correlated with relative $D_{98\text{\%}}$ (ρ=−0.80, p<0.001).

## 4 Discussion

This study demonstrated that accurate real-time tumor tracking on 2D cine-MRI is feasible with a promptable foundation model, even with limited labeled data. Methods A and B showed no significant performance differences, with Method A yielding slightly more favorable geometric metrics and lower runtime. Temporal errors were generally brief, with sustained boundary inaccuracies more common than substantial centroid errors.

Comparison with the next-best official submissions highlights different performance profiles (Table 1). The second-ranked method used a fine-tuned SAM2-based pipeline and achieved the highest relative $D_{98\text{\%}}$ while remaining close to the top geometric performance. The third-ranked method used a training-free point-tracking strategy based on CoTracker [20], achieving the fastest runtime but lower overlap and boundary accuracy than the two top-ranked SAM2/MedSAM2-based approaches. These profiles illustrate trade-offs between geometric accuracy, relative $D_{98\text{\%}}$, and runtime.

The observed performance was numerically stronger than values reported for deformable-registration and template-matching approaches in prior MR-guided studies [3], [6], although these cross-study comparisons should be interpreted cautiously since no direct baseline was evaluated.

These findings are consistent with recent MRgRT literature. Foundation-model pipelines using promptable video segmentation have shown strong tracking performance with limited per-patient supervision [21]. Patient-specific adaptation has also been proposed [19], but its benefit should be interpreted in relation to interobserver variability. Once performance approaches the variability between expert annotations, further improvements against a single reference mask may partly reflect closer reproduction of one observer’s annotation style rather than a clinically meaningful improvement. The TrackRAD2025 challenge report similarly noted that the best-performing submissions achieved performance comparable to interobserver variability [8], consistent with the small differences observed between Method A and Method B. Patient-specific adaptation therefore remains relevant for reducing failure modes or improving temporal stability, but should not be assumed to improve average accuracy beyond the interobserver variability range.

This study inherits limitations from the TrackRAD2025 challenge design. First, the labeled training set was small relative to the variability of anatomies, scanners, and acquisition settings, which may limit generalizability in clinical practice. Second, the task was defined on single-plane sagittal cine-MRI and assumed target visibility within the imaging plane, whereas clinical workflows may involve through-plane motion and intermittent visibility. Third, the surrogate dose metric approximates MLC tracking using centroid-shifted dose distributions and captures only part of the dosimetric impact of segmentation errors. Its strong association with centroid error was therefore expected, while the dosimetric relevance of boundary inaccuracies could not be determined. The metric does not model target deformation, organ-at-risk dose, treatment latency, plan modulation, or full dose delivery and should not be interpreted as evidence of improved clinical dose delivery.

In this setting, the pseudo-label-augmented Method B pipeline did not yield a clear advantage. Although Method B incorporated 296 pseudo-labeled sequences, the ensemble did not provide a statistically significant advantage over Method A, in contrast to our expectations. Because Method B also differed in ensembling, splits, and temporal subsampling, this does not isolate the pseudo-label effect, but indicates that the complete Method B pipeline, despite the added supervision, provided no measurable gain. Possible explanations include residual pseudo-label noise, difficulty identifying the correct target, reinforcement of teacher-specific error patterns, and limited additional motion diversity from temporal subsampling—although these were not tested directly.

Overall, the ablation results suggest that task-specific fine-tuning was the main contributor to the performance gain over zero-shot MedSAM2 propagation. Checkpoint averaging should therefore be interpreted as a prospective challenge-time consolidation strategy that preserved the performance of the best selected checkpoint in a single deployable model, rather than as a source of measurable hidden-test improvement. Ensembling may become more effective as pseudo-label quality improves or more labeled data becomes available, but in the present study it did not demonstrate statistically significant superiority over the single-model approach, which was preferable because of its lower runtime and complexity.

## CRediT authorship contribution statement

**Amparo S. Betancourt Tarifa:** Writing – original draft, Visualization, Validation, Software, Investigation, Formal analysis, Conceptualization. **Marcel Verheij:** Writing – review & editing, Supervision. **René Monshouwer:** Writing – review & editing, Supervision. **John J. Hermans:** Writing – review & editing, Supervision. **Peter J. Koopmans:** Writing – review & editing, Supervision, Conceptualization. **Erik van der Bijl:** Writing – review & editing, Supervision, Conceptualization.

## Code availability

Code and model weights relevant to this study are available on Zenodo [15].

## Ethics declaration

The authors have NOT obtained informed consent from participants or their legal representatives. The present study used previously collected, de-identified data from the TrackRAD2025 dataset and involved no new participant recruitment, intervention, or data collection. Therefore, additional study-specific informed consent was not required for this secondary analysis. For the original data collection, ethical approval was obtained from the relevant institutional review boards or medical ethics committees of the contributing institutions, and patient consent was handled in accordance with applicable local regulations.

This study was performed in compliance with relevant laws, regulatory frameworks and guidelines where the research took place. Ethics committee approval was not required under relevant laws and institutional guidelines. This study used previously collected, de-identified data from the TrackRAD2025 dataset and involved no new participant recruitment, intervention, or data collection. Therefore, no additional ethics committee approval or study-specific informed consent was required for this secondary analysis. The original data collection and use were approved by the relevant institutional review boards or medical ethics committees of the contributing institutions and conducted in accordance with applicable local regulations.

## Declaration of generative AI and AI-assisted technologies in the manuscript preparation process

During the preparation of this work, the authors used ChatGPT in order to improve the readability and language of the manuscript. After using this tool, the authors reviewed and edited the content as needed and take full responsibility for the content of the published article.

## Funding

Elekta AB provided financial support for part of the work presented in this manuscript (Statement of Work ID: RUMC2022DL).

## Declaration of competing interest

The authors declare the following financial interests/personal relationships which may be considered as potential competing interests: Amparo S. Betancourt Tarifa reports financial support was provided by Elekta AB. If there are other authors, they declare that they have no known competing financial interests or personal relationships that could have appeared to influence the work reported in this paper.

## Data Availability

The TrackRAD2025 dataset is publicly available [11].
